# Antiparasitic Effects of Potentially Toxic Beetles (Tenebrionidae and Meloidae) from Steppe Zones

**DOI:** 10.3390/toxins13070489

**Published:** 2021-07-14

**Authors:** Marta Díaz-Navarro, Paula Bolívar, María Fe Andrés, María Teresa Gómez-Muñoz, Rafael A. Martínez-Díaz, Félix Valcárcel, Mario García-París, Luis M. Bautista, Azucena González-Coloma

**Affiliations:** 1 Museo Nacional de Ciencias Naturales, CSIC, José Gutiérrez Abascal 2, 28006 Madrid, Spain; maartadn@gmail.com (M.D.-N.); pbolivar@mncn.csic.es (P.B.); mparis@mncn.csic.es (M.G.-P.); lm.bautista@csic.es (L.M.B.); 2 Instituto de Ciencias Agrarias, CSIC, Serrano 115-dpdo, 28006 Madrid, Spain; mafay@ica.csic; 3 Facultad de Veterinaria, Universidad Complutense de Madrid (UCM), 28040 Madrid, Spain; mariateresa.gomez.munoz@pdi.ucm.es; 4 Facultad de Medicina, Universidad Autónoma de Madrid (UAM), Arzobispo Morcillo S/N, 28029 Madrid, Spain; rafael.martinez@uam.es; 5 Grupo de Parasitología Animal, Departamento de Reproducción Animal, CSIC-INIA, 28040 Madrid, Spain; valcarcel.felix@inia.es

**Keywords:** Tenebrionidae, Meloidae, nematicide, antiprotozoal, GCMS, cantharidin, ethyl oleate, otididae

## Abstract

Arthropods and specifically beetles can synthesize and/or sequester metabolites from dietary sources. In beetle families such as Tenebrionidae and Meloidae, a few studies have reported species with toxic defensive substances and antiparasitic properties that are consumed by birds. Here we have studied the antiparasitic activity of extracts from beetle species present in the habitat of the Great Bustard (*Otis tarda*) against four pathogen models (*Aspergillus niger*, *Meloidogyne javanica*, *Hyalomma lusitanicum*, and *Trichomonas gallinae*). The insect species extracted were *Tentyria peiroleri*, *Scaurus uncinus*, *Blaps lethifera* (Tenebrionidae), and *Mylabris quadripunctata* (Meloidae). *M. quadripunctata* exhibited potent activity against *M. javanica* and *T. gallinae*, while *T. peiroleri* exhibited moderate antiprotozoal activity. The chemical composition of the insect extracts was studied by gas chromatography coupled with mass spectrometry (GC-MS) analysis. The most abundant compounds in the four beetle extracts were hydrocarbons and fatty acids such as palmitic acid, myristic acid and methyl linoleate, which are characteristic of insect cuticles. The presence of cantharidin (CTD) in the *M. quadripunctata* meloid and ethyl oleate (EO) in *T. peiroleri* accounted for the bioactivity of their extracts.

## 1. Introduction

Arthropods contain secondary metabolites (defensins and toxins) characterized by great chemical and biosynthetic diversity [1]. Specifically, coleopterans produce a wide variety of active compounds or metabolites that are medicinal substances or precursors [2,3]. Other Coleoptera acquire toxic substances by sequestering secondary metabolites from dietary sources, which are then transferred from invertebrates to upper trophic levels within the ecosystem [1,4,5]. 

The consumption of secondary metabolites by animals can affect their parasite and pathogen load, regardless of their toxicity to the animal itself. These effects are of particular interest because there is an urgent need to search for natural products with therapeutic potential due to increasing resistance to conventional animal and plant antiparasitic products [6,7]. For example, different phyla of marine organisms are a rich source of bioactive metabolites with biotechnological potential, effective as antiprotozoal agents (algal extracts) [8,9], antitumor agents (terpenoids in cnidarians) and useful for the production of therapeutic products against HIV, inflammatory conditions and microbial diseases [10]. The pharmacological activity of certain terrestrial invertebrates has also been described [11,12,13], but such studies are very limited. 

Toxic coleopterans forming part of animal diets could have antiparasitic activity over and above their nutritional value. However, the medicinal functions of toxic metabolites present in dietary insects are difficult to demonstrate in wild animals [14,15]. However, indirect evidence can be obtained by analyzing the antiparasitic effects of their extracts and metabolites. A recent example is the Great Bustard (*Otis tarda*), a steppe bird present in the Iberian Peninsula that consumes the toxic meloid beetle *Berberomeloe majalis* [12,16]. Blister beetles of the family Meloidae secrete cantharidin (CTD) are considered a sexual stimulant with medicinal applications [17,18] and are highly toxic to most vertebrates [12,18,19,20,21]. The intentional ingestion of these beetles could reduce the parasite load of *O. tarda* since both CTD and extracts of these meloids exhibit nematicidal, antiprotozoal, and bactericidal activity at low concentrations, and lower potency ixodicidal effects [12,16,20]. 

Other potentially toxic Coleoptera species of the Meliodae and Tenebrionidae family are ingested by *O. tarda*. The family Tenebrionidae, commonly called darkling beetles, are protected by chemicals produced in large glands. The chemical constituents of their defensive secretions contain hepatotoxic benzoquinones and long-chain 1-alkenes [3]. Extracts from defensive secretions along with some of the individual compounds have exhibited several pharmacological effects, including cytotoxicity, anti-diabetes, anticoagulatory, and anti-inflammatory [3]. However, little is known of their antiparasitic effects or their composition. 

As part of a broad ongoing study on the diet-based self-medication behavior of the Great Bustard, we analyzed the antiparasitic potential of four species of Coleoptera (Families Tenebrionidae, and Meloidae) distributed in the same areas of the Great Bustard against representative parasite models (nematode, *Meloidogyne javanica*; protozoa, *Trichomonas gallinae*; fungi, *Aspergillus niger,* and ectoparasite, *Hyalomma lusitanicum*).

## 2. Results

**Figure 1:**
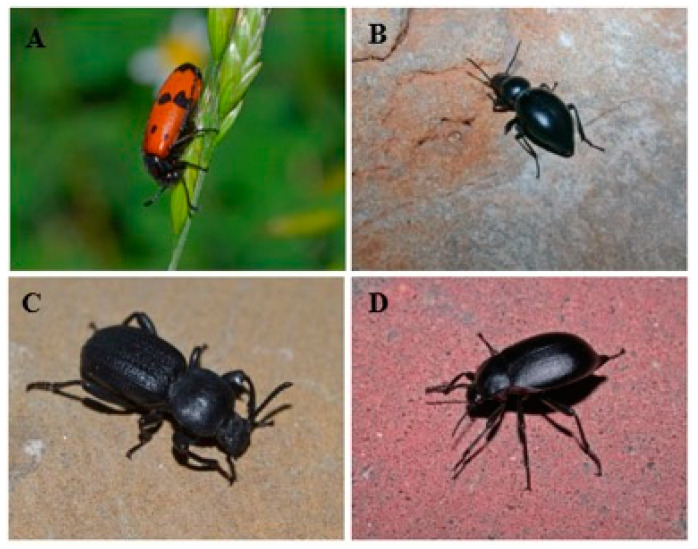
The four beetle species (Coleoptera) studied: *Mylabris quadripunctata* (Linnaeus, 1767) (**A**), *Tentyria peiroleri* (Solier, 1835) (**B**), *Scaurus uncinus* (Forster, 1771) (**C**), and *Blaps lethifera* (Marsham, 1802) (**D**). (Photographs: M. García-París).

Four coleopteran species (three Tenebrionidae and one Meloidae, Figure 1) were collected in the area of distribution of the Great Bustard (*Otis tarda*) and extracted with dichloromethane (DCM). 

Each beetle species yielded varying amounts of organic extracts. The highest yield was obtained from *Blaps lethifera* (22.14%), the other three species yielding less than 10% (Table 1).

The antiparasitic activity of the extracts against two models: a nematode (*Meloidogyne javanica*) and a protozoan (*Trichomonas gallinae*), is shown in Table 2 and Table 3. The extract of the beetle *M*. *quadripunctata* exhibited high activity against the nematode and protozoan models, while *T*. *peiroleri* had moderate antiprotozoal effects. No bioactivity was observed for the other two tenebrionid extracts.

The most active extract against *T. gallinae* was from *M. quadripunctata* (100% at the highest concentration), followed by *T. peiroleri* (66% inhibition at the highest dose). None of the other extracts were trichomonacidal (Table 3).

None of the four coleopteran extracts exhibited noteworthy activity against the ectoparasite model *Hyalomma lusitanicum* or the fungus model *Aspergillus niger* (Table 4).

The chemical profiles of beetle extracts studied by GC-MS (Table 5) showed qualitative and quantitative variations in composition, with a high content of alkanes and fatty acids. The latter were identified as palmitic or hexadecanoic acid, myristic or tetradecanoic acid and methyl linoleate or 9,12,15-octadecatrienoic acid-methyl ester. Muskolactone was present in *T. peiroleri* and *S. uncinus* extracts and muskolactone-related compounds in all the extracts analyzed. *B. lethifera* contained 1-tridecanol. The active extracts were differentially characterized by the presence of CTD in the meloid, *M. quadripunctata* and ethyl oleate (EO) in the tenebrionid, *T*. *peiroleri*.

Pure CTD and EO were tested against *T. gallinae*. CTD showed a strong inhibition of protozoan growth (Table 6) at lower concentrations than the extract, while ethyl oleate was moderately trichomonacidal with activity levels close to those of the extract (Table 6)

## 3. Discussion

This is the first report on the antiparasitic activity of extracts from the Tenebrionidae species *Tentyria peiroleri* and the Meloidae *Mylabris quadripunctata* against the nematode *Meloidogyne javanica* (*M. quadripunctata*) and the protozoan *T. gallinae* (both insect species). These results are similar to those observed for a DCM extract of the toxic meloid beetle *Berberomeloe majalis* [12], which showed nematicidal activity against *M. javanica* but with lower potency than the *M. quadripunctata* extract tested in this study. The *B. majalis* extract also showed moderate ixodicidal effects against *H. lusitanicum*, insect (aphid) antifeedant effects and trichomonacidal activity against *T. vaginalis* [12].

The consumption of this toxic insect by *Otis tarda* has been attributed to possible self-medication behavior [19]. Two of the beetle taxa studied here form part of the Great Bustard diet (*Mylabris* and *Tentyria*, [22]). The other two taxa are available in their habitats (*Scaurus* and *Blaps*) but are not part of their diet [22]. Some species of the genus *Mylabris* used in folk medicine showed potent anti-cancer activity related to their content in cantharidin and related analogs [3]. Similarly, some *Blaps* species have a long history of use in folk medicine attributable to the significant enhancement of the phagocytic ability of mammal macrophages by beetle polysaccharides [23] and the presence of benzoquinones in their defensive secretions along with antimicrobial fatty acids (mainly octadecanoids) of the cuticle [24].

The chemical profiles of the beetle extracts revealed a high content of alkanes and fatty acids. Alkanes are common hydrocarbons in the cuticle of insects [24,25,26]. Among the fatty acids identified, palmitic is a common metabolite of Coleoptera such as *Ulomoides dermestoides* (family Tenebrionidae), and myristic acid and methyl linoleate are among the most abundant cuticular compounds in insects [26], thus accounting for the presence of these compounds in the extracts studied. In addition to acting as a barrier against microorganisms or to prevent desiccation, the involvement of some cuticular hydrocarbons and fatty acids in pheromonal processes has also been described [27]. In arthropods, cuticular hydrocarbons function as intra- and interspecific recognition signals providing behavioral responses in social species. They have also been identified in these insects as chemical signals responsible for maintaining the status of each individual within the hierarchy [28]. They are even necessary for gender recognition, examples being tsetse flies and cerambycid beetles in which males and females possess distinctive hydrocarbons not produced by the opposite gender [27,29,30]. Additionally, muskolactone was present in *T. peiroleri* and *S. uncinus* along with muskolactone-related compounds in all the extracts analyzed. Muskolactone is an aroma used in the perfume industry as it is considered an aphrodisiac but has no reported antiparasitic effects [31].

**Figure 2:**
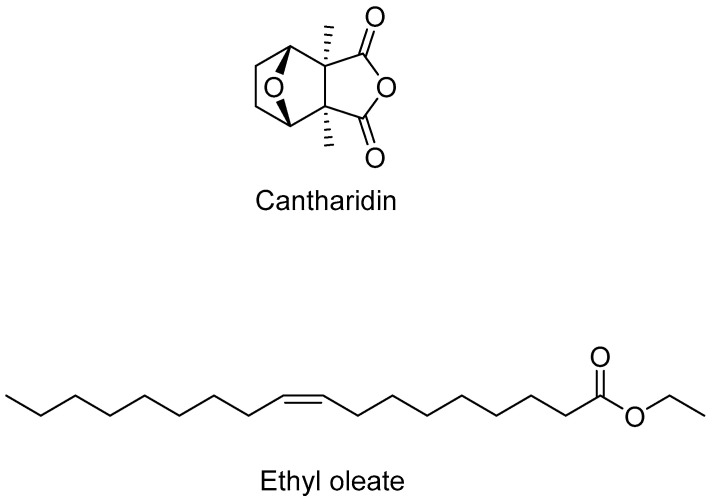
Chemical structure of cantharidin (CTD) and ethyl oleate (EO).

The active extracts were characterized by the presence of cantharidin (CTD) in the meloid, *M*. *quadripunctata* a toxic trycyclic monoterpene (3,6-epoxy-1,2-dimethylcyclohexane-1,2-dicarboxylic anhydride), and ethyl oleate (EO), a fatty acid ester formed by the condensation of oleic acid and ethanol in the tenebrionid, *T. peiroleri* (Figure 2).

The presence of CTD in *M. quadripunctata* has already been described [32]. The identification of CTD in the defensive secretions of beetles of the family Meloidae and Oedemeridae (Coleoptera) is relatively well studied [21,33], with a great variability reported in the amount of CTD between and within meloid species [12,16,19,34].

Ethyl oleate (EO), identified in the antiprotozoal extract of *T*. *peiroleri*, plays an important role in the sexuality of some bees [35] and has been described as a pheromone responsible for inducing arousal behavior in males of *Megachile rotundata* when emitted by females [25]. EO also has antimicrobial effects [36], and acaricidal, repellent, and oviposition deterrent actions against *Tetranychus cinnabarinus* [37].

Some Coleoptera of the family Tenebrionidae have defensive glands that secrete toxic mixtures of metabolites such as the benzoquinones described in *Blaps femoralis* [24] and *B. nitens laportei* [38]. However, benzoquinones were not detected in *B. lethifera*, most likely because the extract was obtained from whole insects rather than from glandular discharges [24,38].

Both compounds, CTD and EO, were further tested against *T. gallinae* with CTD showing high trichomonacidal activity followed by EO with moderate but significant effects. CTD is effective against bacteria such as *Bacillus* sp., *Clostridium* sp., and *Kocuria* sp. [16], and it has been reported as trichomonacidal against the human parasite *Trichomonas vaginalis*, with an LD_50_ slightly lower than the one calculated here for the bird parasite *T*. *gallinae* (5.6 vs. 8.5 µg/mL) [12]. This is the first report on the trichomonacidal effects of EO. The trichomonacidal activity observed for the meloid *M*. *quadripunctata* and the tenebrionid *T*. *peiroleri* extracts can be explained by their content in CTD and EO respectively. Additionally, CTD has been reported as being a strong nematicidal against *M*. *javanica* (LD_50_ of 0.252, and an LD_90_ of 0.065 µg/µL) [12], explaining the nematicidal effects of the *M*. *quadripunctata* extract. CTD is also moderately ixodicidal against *H. lusitanicum* (LD_50_ of 12.84 and LD_90_ of 20.31 µg/mg) [12]. Therefore, the lack of ixodicidal effects of the CTD-containing extract of the meloid, *M*. *quadripunctata* could be the due to its low CTD concentration.

The results of this study contribute to enhancing the knowledge of zoopharmacognosy and the investigation of natural products with potential for use in both veterinary and botanical pharmacology. Numerous studies have shown the existence of self-medication behavior in vertebrates [39,40,41,42,43] and invertebrates [44,45,46]. The fact that the most active extracts studied here are from coleopteran species found in the Great Bustard diet while the least active ones are not, suggests a self-medication function for the toxic compounds present in these insect species. However, future studies on the effects of the coleopteran extracts/compounds on pathogens from wild animals are needed. Furthermore, *Blaps* and *Scaurus* sp have active defensive behavior against predators [47,48] and release a stream of benzoquinones [49,50], while *Mylabris* and *Tentyria* do not show any active defense behavior. An active defense activity along with a low self-medication function could explain why *Blaps* species are not found in Great Bustards diet.

An additional, non-mutually exclusive explanation of the presence/absence of these insects in the Great Bustards diet is circadian beetle behavior: *Blaps* and *Scaurus* are primarily nocturnal while *Mylabris* and *Tentyria* are diurnal. Great Bustards exhibit some nocturnal activity outside of cold winter nights. For example, Great Bustards can be active during darkness in Spring [51] and encounters with the nocturnal beetle species cannot be ruled out, but this possibility is speculative because Great Bustards are diurnal foragers [52]. Nocturnal beetles, therefore, are rarely found in the diet of daytime foragers.

## 4. Conclusions

In this study, dose-dependent nematicidal and trichomonicidal effects of *Mylabris quadripunctata* and *Tentyria peiroleri* extracts have been found. The nematicidal effect against *Meloidogyne javanica* and the antiprotozoal effect against *Trichomonas gallinae* of *Mylabris quadripunctata* extract is related to the presence of active cantharidin in this blister beetle. A moderate antiprotozoal effect of *Tentyria peiroleri* extract has also been observed and can be attributed to the presence of active ethyl oleate in the extract. These insects are present in the area of the Great Bustard and could be part of a self-medication function of the compounds present in these toxic species.

## 5. Materials and Methods

### 5.1. Biological Material

Four species of Coleoptera belonging to families in which biosynthesis of defensive toxins has been described, were selected. Three of them belong to the family Tenebrionidae: *Tentyria peiroleri*, *Scaurus uncinus* and *Blaps lethifera*, and one to the family Meloidae: *Mylabris quadripunctata* (Figure 1). The specimens were collected between July and November 2019 in the Iberian Peninsula through direct sampling in areas with the current or potential presence of Great Bustards and other birds. The locations were as follows: Losar de la Vera, Cáceres (40°06′37.06″ N, 05°36′29.85″ W; 24 individuals of *M. quadripunctata*); Villamayor de Santiago, Cuenca (39°45′12.6″ N, 2°56′08.2″ W; 33 individuals of *T. peiroleri*); Cerecinos de Campos, Zamora (41°54′08″ N, 5°28′25″ W, 3 individuals of *B. lethifera*); and Castrogonzalo, Zamora (41°59′22″ N, 5°35′53″ W, 8 individuals of *S*. *uncinus*). The beetles were preserved separately in Falcon tubes at −80 °C while awaiting further processing.

### 5.2. Beetle Extracts

The beetles (complete insects) were homogenized in a mortar with 15 mL dichloromethane (DCM) to obtain the extracts. They were then sonicated for 5 min and filtered under vacuum with a Buchner funnel. This process was repeated two more times with 15 mL DCM to extract as many compounds as possible from the biological material. The filtrate was brought to dryness using a rotary evaporator to calculate the yield (% extract on dry weight basis). The extracts were stored in vials and kept at 4 °C and used within 24 h.

### 5.3. Bioassays

To evaluate the bioactivity of extracts from *T. peiroleri*, *S. uncinus*, *B. lethifera* and *M. quadripunctata*, four biological models were selected to represent four large groups of parasites: fungi, nematodes, ticks, and protozoa. The choice of models was based on experience in handling them in the laboratory as experimental models. The fungal parasite model used was *Aspergillus niger*, a species that belongs to one of the most widely distributed genera of endophytic fungi and a pathogen responsible for aspergillosis in animals, including birds [53,54]; *Meloidogyne javanica*, an obligate endoparasite of plant species [55,56]; *Hyalomma lusitanicum*, a parasite of wild ungulates, was used as the ixodid model [57,58]; and as a protozoan model *Trichomonas gallinae*, a common parasite of avian hosts, especially columbiformes and raptors [59].

#### 5.3.1. Nematicidal Activity

The nematicidal efficacy of the beetle extracts was tested against *Meloidogyne javanica*, an endoparasitic plant nematode species. This parasite was selected as a model organism because of its availability in the laboratory. Therefore, the results are an initial approximation of the sort of anthelmintic activity that could be expected. The *M. javanica* population was continuously maintained on tomato plants (*Solanum licopersicum* L.) grown at 23 ± 1 °C and >70% relative humidity (RH). Two months after plant infestation, *M. javanica* egg masses were collected from root nodules. The egg masses were deposited in filters with distilled water at the bottom for 24–48 h to favor eclosion and larval migration from which we obtained second-stage juveniles (J2s), the only infective stage of the nematode. Juveniles were suspended in 100 mL of distilled water.

Extracts were prepared at a final concentration of 1 μg/μL dissolved in DMSO-Tween 20 (0.6% Tween 20 in DMSO, DMSO-T). Assays were performed in a 96-well flat-bottom plate, as described in Andrés et al. [60], and replicated four times. Each replicate contained approximately 100 nematodes in 95 μL of distilled water and 5 μL of extract or 5 μL of DMSO-T control solution. The plates were incubated in darkness at 23 ± 1 °C for 72 h. After this period, the dead juvenile *M. javanica* were counted with a binocular microscope.

Results are expressed as mortality percentage of infective juveniles corrected for control mortality according to the Schneider-Orelli formula [61,62]:

% MC = ((% MT − % Mc)/(100 − % Mc)) ∗ 100,(1)

where MC is the nematode mortality corrected for the mortality of the control; MT is the mortality observed in the extract, and Mc is the mortality in the DMSO-T control.

For active extracts (mortality >90%), serial dilutions were carried out to determine the effective lethal dose (LD_50_ and LD_90_) by Probit analysis with the software package StatGraphics Centurion XVI 16.1.02. Thymol (Sigma Aldrich) was used as a positive control with LD_50_ and LD_90_ values of 0.14 and 0.25 mg/mL, respectively.

#### 5.3.2. Antiprotozoal Activity

The protozoan model used was *Trichomonas gallinae* isolated from a wood pigeon (*Columba palumbus*) at the facilities of Grupo de Rehabilitación de Fauna Silvestre (GREFA) in Madrid. The trichomonads were maintained in sterile tubes with 5 mL trypticase—yeast extract—maltose (TYM) medium in an incubator at 37 °C with serial passages to regenerate the culture medium every 48 h. Bioassays were performed 48 h after the pass, when the trichomonads were at logarithmic growth phase. First, protozoa were counted in a Neubauer chamber and a concentration of 5 × 105 *T. gallinae*/mL was prepared. Aliquotes of 150 μL per well were inoculated in a 96-well U-bottom plate.

Extracts were prepared at a final concentration of 400 μg/mL and two more dilutions were performed (200 and 100 μg/mL). For pure CTD, the starting concentration was 100 μg/mL and five more dilutions were employed (50, 25, 10, 5, and 1 μg/mL). The activity of each dilution was tested in triplicate. Three controls and four replicates of each were used: a positive control of 10 μL/mL metronidazole, a negative control (the TYM culture medium with the trichomonads), and a blank (the TYM culture medium). These three controls ensured the validity of the test and enabled us to correct the results with the absorbance value that the staining of the medium could provide.

The plate was kept in dark conditions at 37 °C for 24 h. The plate was centrifuged at 2500 rpm at 25 °C for 5 min to precipitate the trichomonads at the bottom of the well so as to easily remove the medium. Once the medium was discarded, 90 μL of sterile phosphate buffered saline (PBS) was added to each well, including 10 μL of 1.25 mg/mL MTT + 0.1 mg/mL phenazine methosulphate (PMS) solution. Wells were incubated for 30 min at 37 °C in the dark. Subsequently, 50 μL of sodium dodecyl sulphate (SDS) was added per well and the plate was incubated again for 15 min at 37 °C in the dark. Finally, absorbance was measured at 620 nm in a spectrophotometer. The following formula was used to calculate the inhibition percentage from the absorbance:

% AT = 100 − ((Ap − Ab)/(Ac − Ab)) ∗ 100,(2)

where % AT corresponds to the inhibitory activity of the extract after subtracting the absorbance of the culture receiving the extract (Ap, absorbance of the test substance) from the absorbance of the blank (Ab), and dividing by the difference between the absorbances of the negative control (Ac) and the blank (Ab). The positive control metronidazole gave an LD_50_ value of 0.6 µg/mL.

#### 5.3.3. Fungicidal Activity

The fungicidal activity of the beetle extracts was evaluated against the fungus *Aspergillus niger*. A suspension of 7.5 × 105 spores/mL in 0.9% NaCl saline (Fisher Scientific, Madrid, CAM, Spain) was prepared using a PDA plate (Difco) from a 3-day culture of *A. niger*.

Extracts were assayed at a concentration of 800 μg/mL dissolved in 1% dimethyl sulfoxide (DMSO). The assays were replicated four times with amphotericin B at 5 μg/μL (Thermo Scientific, Madrid, CAM, Spain) as positive control and 1% DMSO and milli-Q water as negative controls.

Sterile 96-well flat-bottomed plates were used for the assay. Each well was inoculated with an aliquot of 80 μL of corresponding extract, 100 μL of Roswell Park Memorial Institute (RPMI, Sigma-Aldrich, Madrid, CAM, Spain) with 3-N-morpholino propanesulfonic acid (MOPS, Acros Organics) as buffer and 20 μL of the spore suspension. The plates were kept at 28 ± 1 °C in darkness for 24 h. A colorimetric method was used to read results, 25 μL of RPMI solution containing 3-(4,5-dimethylthiazol-2-yl)-2,5-diphenyltetrazole bromide (MTT, Acros Organics) 5 mg/mL, and 0.5–1 mg 1 mM menadione (Acros Organics, Madrid, CAM, Spain) was added to each well and the plate was kept at 28 ± 1 °C in dark conditions for 3 h. After this period, the culture medium was removed by aspiration and 200 μL of acidic isopropanol (95% acidic isopropanol 5% 1 M HCl) was added to each and kept for 30 min at 28 ± 1 °C. The absorbance at 490 nm was measured using an ELISA reader and the Gen5 2.01 reader program.

Germination percentage (%G) was calculated by means of the following formula:

%G = (%GTto/%Gc) ∗ 100,(3)

where %G is the germination percentage of *A. niger*; GTto is the germination of the fungus in the extract test and Gc is the germination observed in the DMSO negative control.

#### 5.3.4. Ixodicidal Activity

*Hyalomma lusitanicum* engorged females were collected from red deer in Ciudad Real (central Spain) and maintained in an incubator (22–24 °C and 80% relative humidity (RH) until oviposition and egg hatching.

Tests were conducted as previously described [63]. Briefly, 50 µL of test solution was added to 25 mg of powdered cellulose at different concentrations (initial concentration of 40 or 20 µg/mg for extracts and pure compounds respectively) and the solvent evaporated. Each test consisted of 20 active larvae, at least 6 weeks old, and was replicated three times. Negative (cellulose) and positive (thymol Sigma Aldrich at 20 µg/mg) controls were also used. The ticks and cellulose were placed in glass tubes and carefully mixed by rotating the tube several times to ensure full tick-cellulose contact and kept in a stove at the conditions described [63].

Dead (not moving) ticks were counted after 24 h with a binocular magnifying glass. Larvicidal activity data are presented as percent mortality corrected according to the Schneider-Orelli formula [62]. Thymol (Sigma-Aldrich) was included as a positive control with LD_50_ and LD_90_ values of 2.94 and 6.16 µg/mg.

### 5.4. Chemical Profiles of Organic Extracts

To determine compounds, extracts were analyzed via GC-MS, using a Shimadzu GC-2010 coupled to a Shimadzu GCMS-QP2010-Ultra mass detector with an electron impact ionisation source at 70 eV and using a Single Quadrupole analyzer with Helium as the carrier gas. Samples were dissolved in 100% DCM and injected by an automatic injector (AOC-20i). Chromatography was carried out with a Teknokroma TRB-5 (95%) dimethyl- (5%) diphenylpolysiloxane capillary column, 30 m × 0.25 mm ID and 0.25 μm phase thickness.

Working conditions were: Split mode injection injecting 1 μL of sample with a split ratio (20:1) using a Shimadzu AOC-20i automatic injector; the injector temperature was 300 °C, the temperature of the transfer line connected to the mass spectrometer was 250 °C and the temperature of the ionisation source was 220 °C. The initial column temperature was 110 °C, heated up to 290 °C at 7 °C/min, and staying at 290 °C for 20 min. The mass spectra and retention time for each peak recorded on the chromatograms were used to identify the compounds by comparison with those found in the Wiley database (Wiley 275 Mass Spectra Database, 2001) and NIST17 (NIST Mass Spectra Database, 2017).

## Figures and Tables

**Table 1:** Number of individuals collected, fresh weight, weight of extract obtained, and yield (g extract/g fresh sample) of the extracts of the four coleopteran species analyzed.

Family	Species	N°	Fresh Weight (g)	Extract Weight (mg)	Yield (%)
Tenebrionidae	*Tentyria peiroleri*	33	4.96	100.70	2.03
	*Scaurus uncinus*	8	1.45	140.90	9.72
	*Blaps lethifera*	3	2.43	537.90	22.14
Meloidae	*Mylabris quadripunctata*	24	2.74	78.50	2.87

**Table 2:** Activity of coleoptera extracts on juvenile mortality in the parasitic nematode *Meloidogyne javanica*.

Coleoptera		µg/µL	Mortality ^a^ %	Lethal Concentrations ^b^	
Family	Species			LC_50_ µg/µL	LC_90_ µg/µL
Tenebrionidae	*Tentyria peiroleri*	1.00	2.00 ± 1.07		
	*Scaurus uncinus*	1.00	0.00		
	*Blaps lethifera*	1.00	0.71 ± 1.03		
Meloidae	*Mylabris quadripunctata*	1.00	97.00 ± 1.00	0.35 (0.33–0.37)	0.65 (0.61–0.69)
		0.50	81.33 ± 1.72		
		0.25	49.00 ± 4.00		
		0.12	7.26 ± 3.38		
		0.06	2.70 ± 1.66		

^a^ Data corrected according to Schneider–Orelli’s formula. Values are the means of four replicates; ^b^ Lethal doses resulted in 50% and 90% mortality (95% Confidence Limits).

**Table 3:** Activity of Coleoptera extracts against the protozoan parasite *Trichomonas gallinae*.

^a^ Data show the percentage of mortality extracted from the mean absorbances ± SE (N = 3); ^b^ Lethal doses resulting in 50% and 90% mortality (95% Confidence Limits).

**Table 4:** Activity of the extracts against the parasitic fungal model *Aspergilllus niger* (% germination inhibition at a dose of 800 µg/mL) and the ectoparasitic model *Hyalomma lusitanicum* (% larval mortality at a dose of 40 µg/mg).

Coleoptera		% Germination Inhibition *Aspergillus niger* ^a^	% Mortality *Hyalomma lusitanicum* ^b^
Family	Species		
Tenebrionidae	*Tentyria peiroleri*	9.52 ± 8.46	5.08 ± 2.89
	*Scaurus uncinus*	21.21 ± 8.69	11.67 ± 1.67
	*Blaps lethifera*	8.65 ± 5.64	7.41 ± 3.73
Meloidae	*Mylabris quadripunctata*	0.00 ± 0.00	3.70 ± 1.91

^a^ Values are the means of four replicates ± SE; ^b^ Values are the means of three replicates ± SE.

**Table 5:** GC-MS analysis of extracts from *Mylabris quadripunctata*, *Tentyria peiroleri*, *Scaurus uncinus* and *Blaps lethifera*. Compound identification, retention time (TR, min), and relative area (%) of the compounds present in the extracts.

Retention Time (min)	Area (%)				Compounds
	*Mylabris quadripunctata*	*Tentyria peiroleri*	*Scaurus uncinus*	*Blaps lethifera*	
3.06	-	-	-	13.34	1-Tridecanol
5.20	2.74	-	-	-	Cantharidin
7.41	1.18	1.72	0.21	-	Myristic acid
9.81	-	1.57	0.41	-	Muskolactone
10.07	9.45	10.32	7.49	7.79	Hexadecanoic acid
12.37	6.29	6.34	3.43	2.83	Methyl linoleate
12.45	21.76	23.22	15.58	10.58	Muskolactone related
12.71	5.03	5.10	2.95	1.91	Octadecanoic acid
12.81	-	1.55	-	-	Ethyl oleate
15.26	2.07	2.29	4.34	6.53	Oleoamide
16.87–35.60	35.58	41.79	61.47	50.76	Alkanes and derivatives

**Table 6:** Activity of ethyl oleate (EO), cantharidin (CTD) against the protozoan parasite *Trichomonas gallinae*.

Treatment	Dose (µg/µL)	Growth Inhibition % ^a^	Effective Concentrations ^b^	
			EC_50_ µg/µL	EC_90_ µg/µL
EO	0.400	66.23 ± 1.57	0.3090 (0.3090–0.3100)	0.5471 (0.5466–0.5476)
	0.200	34.91 ± 1.11		
	0.100	6.85 ± 0.17		
CTD	0.025	97.76 ± 7.12	0.0085 (0.0084–0.0085)	0.0287 (0.0286–0.0287)
	0.010	75.18 ± 5.33		
	0.005	45.63 ± 7.40		
	0.001	0		

^a^ Data show the percentage of growth inhibition extracted from the mean absorbances ± SE (N = 3); ^b^ Lethal doses resulting in 50% and 90% growth inhibition (95% Confidence Limits).

## Data Availability

Data are available upon request and are kept in the laboratory data repository.
